# Tracing the Expression of Resident Quality of Life Policies in Canadian Long-Term Care Settings

**DOI:** 10.1093/geroni/igab046.1428

**Published:** 2021-12-17

**Authors:** Janice Keefe, Pamela Irwin

**Affiliations:** Mount Saint Vincent University, Halifax, Nova Scotia, Canada

## Abstract

Policies favouring safety, security, and order are expressed in preference to those oriented towards person-centred resident quality of life in Canadian long-term care settings. Factors impacting the expression of these latent (under-utilised) rules were uncovered through an analysis of long-term care related policies in four provinces. 84 policies relating to resident quality of life in long-term care were analysed in three sequences, incorporating jurisdictions, policy types, and quality of life domains, over time. The analysis revealed three policy levers: situations–providing explicit and implicit examples of resident oriented quality of life policy suppression in each jurisdiction; structures–identifying which types of policy and quality of life expressions are more vulnerable to dominance by others; and trajectories–confirming the cultural shift towards more person-centredness in Canadian long-term care related policies over time. Although these policies exist, their potentiality remains dormant in the dominant policy discourse, thereby signaling a positive post-pandemic possibility.

